# Electrochemical DNA Biosensors with Dual-Signal Amplification Strategy for Highly Sensitive HPV 16 Detection

**DOI:** 10.3390/s23177380

**Published:** 2023-08-24

**Authors:** Yuxing Yang, Yazhen Liao, Yang Qing, Haiyu Li, Jie Du

**Affiliations:** College of Materials Science and Engineering, Hainan University, Haikou 570228, China; 20085600210065@hainanu.edu.cn (Y.Y.); 21220856000036@hainanu.edu.cn (Y.L.); 20080500210023@hainanu.edu.cn (Y.Q.); 20080500110012@hainanu.edu.cn (H.L.)

**Keywords:** electrochemical biosensor, high-risk human papillomavirus, gold nanoparticles, 3-Aminopropyltriethoxysilane

## Abstract

Cervical cancer is an important topic in the study of global health issues, ranking fourth among women’s cancer cases in the world. It is one of the nine major cancers that China is focusing on preventing and treating, and it is the only cancer that can be prevented through vaccination. Systematic and effective screening for human papilloma (HPV) infection, which is closely linked to the development of cervical cancer, can reduce cervical cancer incidence and mortality. In this paper, an electrochemical sensor was designed to detect HPV 16 using dual-signal amplification. An APTES-modified glassy carbon electrode was used for improved stability. Gold nanoparticles and a chain amplification reaction were combined for signal amplification. The limit of detection (LOD) of this electrochemical sensor was 1.731 × 10^−16^ mol/L, and the linear response of the target detector range was from 1.0 × 10^−13^ mol/L to 1.0 × 10^−5^ mol/L (R^2^ = 0.99232). The test of serum sample recovery showed that it has good anti-interference, and the performance of all aspects was improved to different degrees compared with the previous research from the team. The designed sensor is centered around the principles of low cost, high sensitivity and stability, which provides new ideas for the future development of cervical cancer prevention and electrochemical biosensors.

## 1. Introduction

Cervical cancer is a malignant tumor that poses a serious threat to women’s health [1], and a large number of women around the world tragically pass away from cervical cancer every year. According to studies, human papillomavirus infection (HPV) is one of the main causes of the vast majority of cervical cancers [2]. HPV is a squamous epithelial hyperplasia spherical DNA virus that causes hyperplasia of human skin and mucous membranes [3]. Depending on the level of pathogenicity, HPV is frequently divided into low-risk and high-risk varieties. The main high-risk subtypes are HPV 16, 18, 31, 56, 59 and 68, which cause cervical cancer and cervical intraepithelial neoplasia. Low-risk types include HPV 6, 11, 40, 42, 43, 44, 54, 61, 72, 81 and 89, which primarily cause skin or genital warts [4]. Studies have shown that HPV 16 and HPV 18 have been identified as major causative factors for cervical cancer [5]. It is worth noting that the average age of onset of cervical cancer has been gradually decreasing in recent years, and there is a trend towards a younger age group [6]. Mortality from cervical cancer continues to rise, especially in developing countries where medical facilities are limited. The screening and prevention of this disease are, therefore, urgent. The key to solving these problems lies in rapid means of detection, as well as low-cost, highly accurate and convenient specialized equipment.

The rapid and sensitive detection of specific biomarkers are very important for disease detection. These techniques, such as loop-mediated isothermal amplification (LAMP) [7], polymerase chain reaction (PCR) [8] and rolling circle amplification (RCA) [9], are routinely used to amplify targets to detectable levels [10]. Obviously, there are some drawbacks to these means, such as the need for complex detection equipment, long incubation time and expensive costs. Electrochemical biosensors can achieve high sensitivity compared to conventional detection methods, and they can meet the experimental conditions in a room-temperature environment. The electrochemical biosensors have the ability to monitor in real time and provide results in a short period of time, and the small size and portability of the electrochemical sensors can support a wide range of scenarios. Recently, electrochemical biosensors have been used to detect HPV and other DNA sequences [11]. For example, Liu et al. investigated an electrochemical sensor with signal amplification achieved by signal output through hybridization chain reaction (HCR) to achieve better sensitivity, accuracy and stability [12]. Nucleic acid amplification strategies with electrochemical sensors have been successfully used for nucleic acid detection for nucleic acids [13], small molecules [14] and proteins [15].

The stability of the electrode interface plays a key role in both sensor detection reproducibility and sensitivity. 3-Aminopropyltriethoxysilane (APTES) is a chemical reagent typically used as an intermediate chemical mediator to create a “solid bridge” between a bioprobe and an electrode substrate by surface functionalizing the substrate to form a film [16,17,18]. Modification of the electrode substrate with APTES enables good connection of DNA to the electrode and improves the stability of the sensor [19]. Siddique et al. used APTES to link polydimethylsiloxane (PDMS) and proteins to form an APTES coating, which improved cell stability and proliferation using the effect that APTES can stabilize the matrix within the microchannel [20]. Suparat Cotchim and colleagues designed a biosensor modified with APTES to detect trace DNA, and the amine group of APTES interacts with the DNA phosphate backbone to improve the performance of the sensor [21]. In recent years, functionalization with APTES has been a relatively mature technique and has been successfully applied to detect proteins [22], nucleic acids [23] and bacteria [24].

Here, we made further optimizations based on previous studies [25]. A dual-signal amplified DNA electrochemical biosensor for the detection of HPV 16 was created using APTES modified on the surface of a glassy carbon electrode. In order to prevent the creation of disordered polymers via the hydrolysis of APTES molecules in the system, the performance of the glassy carbon electrode surface was changed with APTES, and the water in the reaction environment was closely regulated. Gold nanoparticles can increase electrode conductivity [26,27,28]. The introduction of gold nanoparticles, which take advantage of the high specific surface area of gold nanoparticles, allows more capture probes to be immobilized on the electrode. AP1 and AP2 are two single strands of DNA with base mismatches complementary to each other, which spontaneously undergo strand amplification in the system for signal amplification of the target detectors. According to the experimental results, the sensor has a low detection limit and a broader detection area, providing a quick and simple method for detecting HPV.

## 2. Materials and Methods

### 2.1. Instruments

A CS350H electrochemical workstation was used to conduct the electrochemical experiments (Hubei Wuhan Crest Instruments Co., Ltd., Wuhan, China). The electrode system used for the experiments was a conventional three-electrode system, with all electrodes procured from Wuhan GOSRELI Technology Co. (Wuhan, China). The electrodes included glassy carbon electrodes with different modifications as working electrodes, platinum wires as auxiliary motors and Ag/AgCl electrodes as reference electrodes. Scanning electron microscopy (SEM) was used to analyze the surface morphology of electrodes by using the Czech Tescan MIRA LMS, transmission electron microscopy (TEM) was performed using FEI Talos 200S (HRTEM, Waltham, MA, USA) for sample morphology analysis and ultraviolet–visible spectrophotometry (UV-Vis) was performed by using PE Lambda 750s (PerkinElmer, Waltham, MA, USA) for sample analysis.

### 2.2. Reagents

6-mercapto-1-hexanol (MCH), potassium ferricyanide (K_3_Fe(CN)_6_), potassium chloride (KCl), magnesium chloride (MgCl_2_) and sodium chloride (NaCl) were purchased from Aladdin Ltd. (Shanghai, China). Sulfuric acid (H_2_SO_4_) and anhydrous ethanol (C_2_H_6_O) were purchased from Xilong Science Co (Guangzhou, China). Tetrachloroauric acid (HAuHCl_4_), 3-aminopropyltriethoxysilane (APTES) and tris(2-hydroxyethyl)phosphine (TCEP) were purchased from Shanghai Maclean Biochemical Technology Co (Shanghai, China). Tris-HCl buffer (pH 7.4) was purchased from Biotech Bioengineering Co. (Shanghai, China) Dichlorotris(1,10-phenanthroline)ruthenium(II) chloride ([Ru(phen)_3_]Cl_2_) was purchased from Sigma Aldrich (Shanghai) Trading Co. Adenosine triphosphate solution (ATP) and TE buffer (pH 7.4, 10 mmol/L Tris-HCl, 1 mmol/L EDTA) were purchased from Shanghai Yuan Ye. Trisodium citrate (C_6_H_5_O_7_Na_3_) is available from Thermo Fisher Scientific. The purity of the purchased chemicals was of analytical purity. Ultra-pure water (18.25 Ω) made from Plus-E3 of Nanjing EPC Technology Development Co., Ltd. (Nanjing, China) was used as the experimental water.

The DNA synthesized by Shanghai Biotechnology Co., Ltd. (Shanghai, China) and purified via HPLC was used in this experiment (Table 1). The DNA was centrifuged first; we then added TE buffer to solubilize it and stored at 4 °C.

### 2.3. Pretreatment of Glassy Carbon Electrode

Prepare the electrode polishing box and moisten the alumina powder poured on the polishing cloth with ultra-pure water to make a paste. Polish the 3 mm diameter glassy carbon electrode with 0.3 μm and 0.05 μm alumina powder in vertical “8” shape until the surface is smooth. After five minutes of sonication in ultra-pure water, the electrode was treated in turn with ethanol and ultra-pure water using the same procedure to remove the aluminum oxide particles from the electrode surface.

Cyclic voltammetry (CV) scanning was used to accomplish electrochemical cleaning in 50 mmol/L H_2_SO_4_ solution until the curve was steady from −0.4 V to +1.6 V at 0.1 v/s. For use in following tests, the electrode was dried using a powerful hairdryer on cold air setting after being rinsed with ultra-pure water.

### 2.4. Glassy Carbon Electrodes Modified with APTES

The reaction vessel was covered with cling film and incubated for 2 h with the electrodes submerged in a 1% ethanolic solution of APTES (*v*/*v*). The electrode was then taken out, promptly placed in a solution of anhydrous ethanol and sonicated for 2 min.

### 2.5. Preparation of AuNPs

Then, 30 mL of 0.01% HAuHCl_4_ solution was loaded into a washed beaker, stirred continuously with a magnetic stirrer and heated to 120 °C in an oil bath. Add 1.5 mL of 1% trisodium citrate solution; 20 min later, stop heating and continue stirring until cooled to room temperature to obtain AuNPs solution, which was stored in a 4 °C environment for backup.

### 2.6. Preparation of CP-AuNPs-MCH via Freeze–Thaw Method

DNA fixation buffer was used, adding 500 mmol/L NaCl to TE buffer (10 mmol/L Tris-HCl, 1 mmol/L EDTA).

Add 6 μL of 10 mmol/L TCEP and 12 μL of 100 μmol/L thiol-modified capture probe (CP), mix well and react for 1 h. Add 400 μL of 1OD (Optical density) AuNPs solution, mix well and freeze in a refrigerator at −20 °C for 2 h. After freezing, thaw at ambient temperature and centrifuge the thawed solution for 20 min at 14,000 rpm. The supernatant was removed with a pipette gun, redissolved in ultra-pure water and repeatedly centrifuged three times to remove excess CP to obtain CP-AuNPs solution.

In order to prevent undesired DNA adsorption, the produced CP-AuNPs solution was centrifuged at 14,000 rpm for 20 min. Discard the supernatant of the solution, dissolve it again in 1 × 10^−8^ mol/L MCH solution and react for 1 h. When the reaction was finished, the mixture was centrifuged for 20 min at 14,000 rpm, we discarded the supernatant of the solution and the process was repeated to eliminate the MCH reaction that had not yet finished. Centrifugation was performed again, and the supernatant was discarded and dissolved in DNA fixation buffer to 1672 μL. Store the prepared CP-AuNPs-MCH solution in a refrigerator at 4 °C.

### 2.7. Preparation of Sensors

DNA hybridization buffer was used, adding 500 mmol/L NaCl and 1 mmol/L MgCl_2_ to TE buffer (10 mmol/L Tris-HCl, 1 mmol/L EDTA).

Thus, add 70 μL of CP-AuNPs-MCH solution to a 2 mL centrifuge tube, place the APTES-modified glassy carbon electrode upside down in the tube, incubate overnight on a shaker at 500 rpm (to prevent the electrode from throwing out too fast, using water bath shaking), rinse the electrode and blow dry at the end of the reaction. On the electrode, 10 μL of 10 mmol/L ATP was injected dropwise and reacted for 1 h. The electrode was blown dry after being cleaned off. The electrode was then washed off and dried after reacting for 2 h with 10 μL of various target DNA (TD) concentrations that were applied dropwise. The electrodes were blown dry after washing and incubated in 10 μL of auxiliary DNA 1 (AP1) species for 1 h. Finally, the combination of 1 μmol/L AP1 and 1 μmol/L auxiliary DNA 2 (AP2) was formed, newly made from hybridization buffer. Then, 10 μL drops were taken onto the electrode and allowed to react for 2 h, then washed off and dried, ready for measurement.

### 2.8. Electrochemical Measurements

In 0.1 mol/L KCl solution that contains 1 mmol/L K_3_Fe(CN)_6_, cyclic voltammetry experiments were carried out. The voltage was between −0.2 V and 0.6 V, and the scan rate was 50 mV/s. In 10 mmol/L Tris-HCl that contained 5 mmol/L [Ru(phen)_3_]^2+^, differential pulse voltammetry measurements were made. The potential was recorded using a range of 0 V to −1 V, a pulse width of 0.05 s, a pulse period of 0.1 s, an amplitude of 50 mV and a potential increment of 1 mV. I_0_ is the peak current in the absence of TD, I_T_ is the peak current in the presence of TD and the signal difference is given as ΔI = I_T_ − I_0_.

## 3. Results and Discussion

### 3.1. Structure of the Sensor

**Figure 1:**
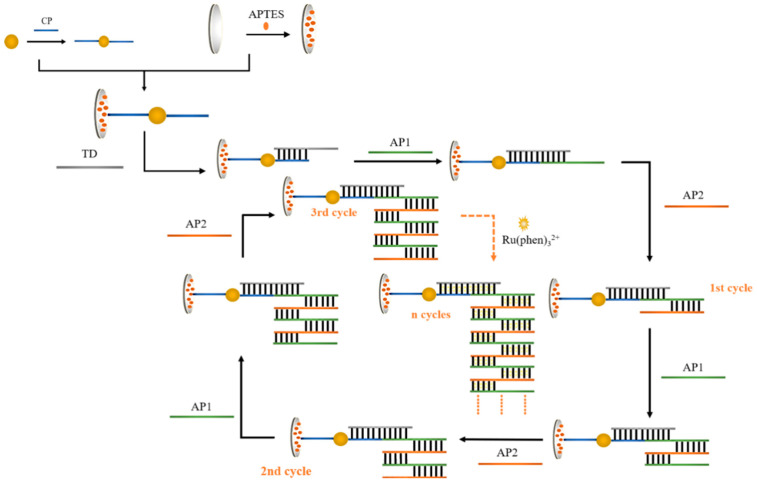
Diagrammatic representation of the dual-signal amplified DNA electrochemical biosensor adapted for APTES.

Figure 1 shows a flowchart of the idea designed for the program. Firstly, HAuHCl_4_ solution and trisodium citrate were used for the preparation of gold nanoparticles, and the sulfhydryl-modified CPs were attached to AuNPs using the freeze–thaw method. The glassy carbon electrode was subjected to covalent bonding modification using APTES ethanol solution. During the process of self-assembly, APTES molecules were fixed on the electrode surface to create an APTES membrane. The CP-AuNPs might be immobilized on the electrode surface via electrostatic interactions between the phosphate groups on the 5 ends of the DNA single strand and the terminal amino groups of the APTES membrane. Subsequently, one end of TD was hybridized with CP through base complementary pairing, and the other end was hybridized with AP1. Since the designed AP1 and AP2 are two single strands of DNA with base mismatch complementarity, they will spontaneously undergo a chain amplification reaction in the system to form a long DNA nanostructure. TD connects this one-dimensional DNA nanostructure to the electrode via hybridization to AP1. Gold nanoparticles have a high specific surface area, which causes more CPs to attach and, in turn, capture more AP1 and AP2 nanostructures. The majority of [Ru(phen)_3_]^2+^ also enters the negatively charged DNA double helix structure through electrostatic interactions, which improves the signal value in electrochemical measurements and enables double-signal amplification.

### 3.2. Electrochemical Characterization

**Figure 2:**
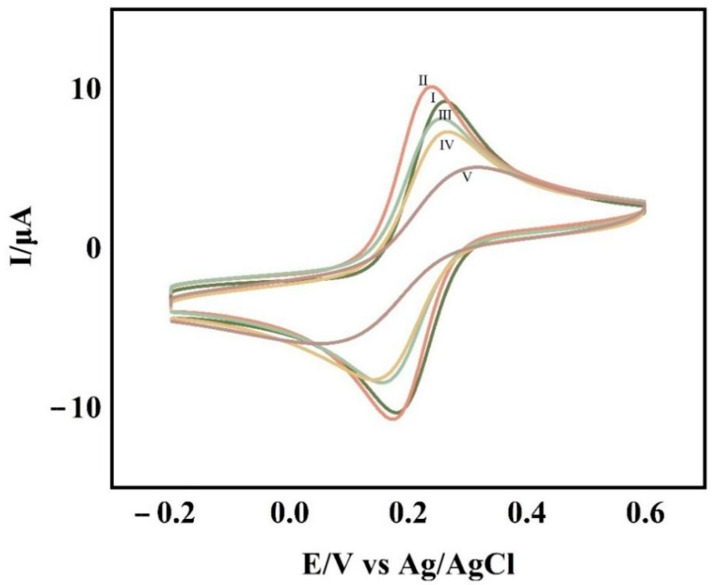
CV at bare GCE (Ⅰ), GCE/APTES (Ⅱ), GCE/APTES/CP-AuNPs (Ⅲ), GCE/APTES/CP-AuNPs/TD (Ⅳ), GCE/APTES/CP-AuNPs/TD/AP1/AP2 (Ⅴ) in 0.1 mol/L KCl solution containing 1 mmol/L K_3_Fe(CN)_6_.

Cyclic voltammetry was used to characterize each step of the experimental operation and was used to determine the completion of each step of the experimental operation. The cyclic voltametric profiles of the glassy carbon electrodes with various degrees of change are shown in Figure 2. The glass carbon electrode modified with the self-assembled APTES film (Figure 2II) had a higher redox peak than the clean glassy carbon electrode (Figure 2I), indicating that the electrode modification was successful. Additionally, the system’s overall electron transfer efficiency was improved due to APTES’ excellent conductivity. When CP-AuNPs (Figure 2III) are immobilized onto the electrode, although the gold nanoparticles are conductive, which increases the electron transfer efficiency, the large number of negatively charged DNA probes attached to the gold nanoparticles makes the electron transfer pathway blocked due to the non-conductive nature of their attached CPs, making the peaks decrease. The DNA double-stranded structure grew as a result of TD hybridization with CPs (Figure 2IV) and the addition of AP1 and AP2 (Figure 2V), both of which, to various degrees, blocked the electron transfer, causing the peaks to progressively decline.

**Figure 3:**
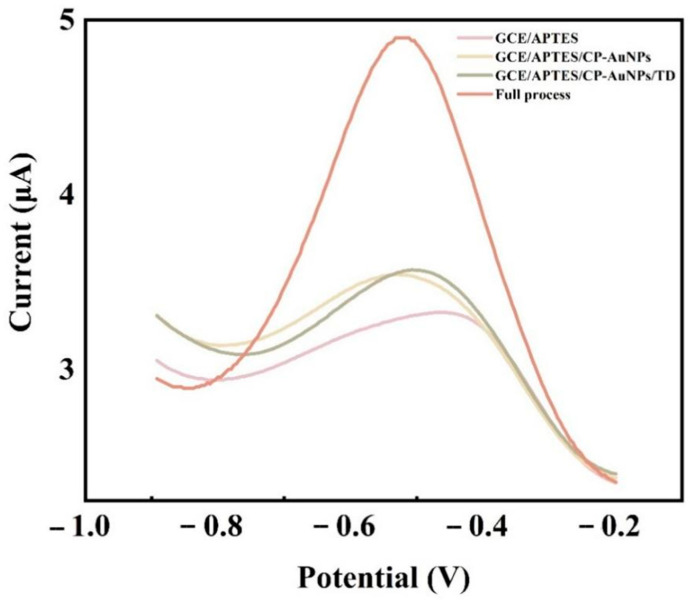
DPV response of oligonucleotide-modified glass carbon electrode in 10 mmol/L Tris-HCl that contained 5 mmol/L [Ru(phen)_3_]^2+^. The concentration of TD is 1 μmol/L, and AP1 and AP2 are both 1 μmol/L as well.

The DPV response signal values of different nucleotide chain modified glassy carbon electrodes are shown in Figure 3. In the context of a glassy carbon electrode modified by APTES molecules, the absence of DNA leaves no way for the signal molecule to bind to the electrode, and, therefore, almost no electrochemical signal values are generated. CP-AuNPs are anchored to the electrode surface by the phosphate group at the 5‘ end of CP binding to the amino group, and since CP is a single chain, [Ru(phen)_3_]^2+^ has low binding to CP, thus producing a low value of current response. The hybridization of CP with TD produced double-stranded DNA, with only a very small amount of [Ru(phen)_3_]^2+^ attached to the double helix structure of DNA, and the signal value increased slightly. After the addition of AP1 and AP2 to the system, the electrochemical signal was greatly enhanced by the embedding of large amounts of [Ru(phen)_3_]^2+^ after base pairing of TD, and AP1 was adsorbed into the double helix structure of the DNA, which immobilized the long-stranded DNA formed by AP2 and AP1 amplification to the electrode surface. The feasibility of the scheme was demonstrated.

### 3.3. SEM Characterization of Electrodes

**Figure 4:**
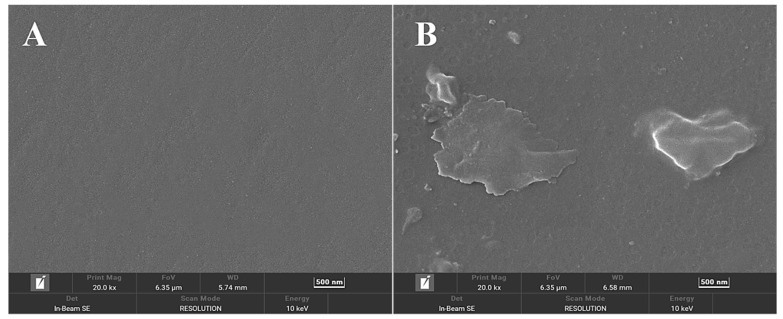
SEM micrographs of (**A**) bare GCE; (**B**) GCE/APTES electrodes.

Analysis of electrode surface morphology was carried out using scanning electron microscopy. The surface layer of the APTES-modified glassy carbon electrode displayed a different villi island shape (Figure 4B) when compared to the naked glassy carbon electrode (Figure 4A). The system is based on the team’s previous scheme [25], which uses a glassy carbon electrode instead of a gold electrode, which has a relatively flat surface with low porosity, limiting the adsorption and coverage of APTES molecules to some extent. The porous structure of the surface of the glassy carbon electrode provides a larger surface area and more adsorption sites for APTES modification. It is not necessary to promote the reaction by increasing the temperature, and the reaction can be carried out at room temperature. By covering the reaction vessel with cling film to stop outside water from entering the reaction system throughout the reaction, the glassy carbon electrode’s surface layer, which included a hydroxylated silica layer, facilitated the modification of APTES simpler and inhibited the hydrolysis of APTES. The organization order of APTES molecules is susceptible to misalignment because of the weak van der Waals interactions between APTES molecules, which results in APTES films with a non-dense internal structure, resulting in a velvety surface.

### 3.4. Characterization of AuNPs

**Figure 5:**
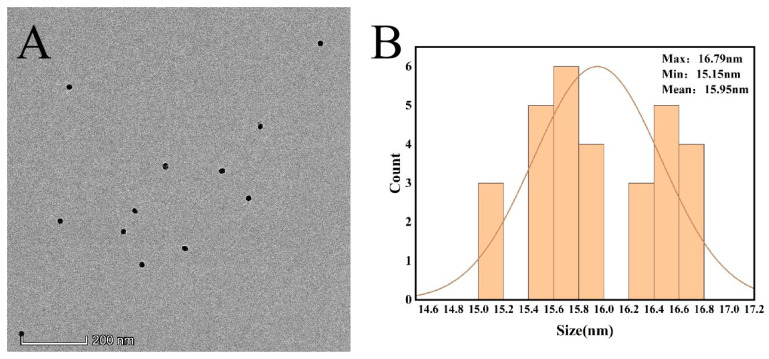
(**A**) TEM image of AuNPs (**B**) particle size distribution of AuNPs.

The morphology of the produced AuNPs was studied using transmission electron microscopy. As can be seen in Figure 5, the AuNPs prepared through this experimental method are spherical structures of uniform size, and the average diameter is 16 ± 1 nm using Nano Measurer software for particle size analysis.

**Figure 6:**
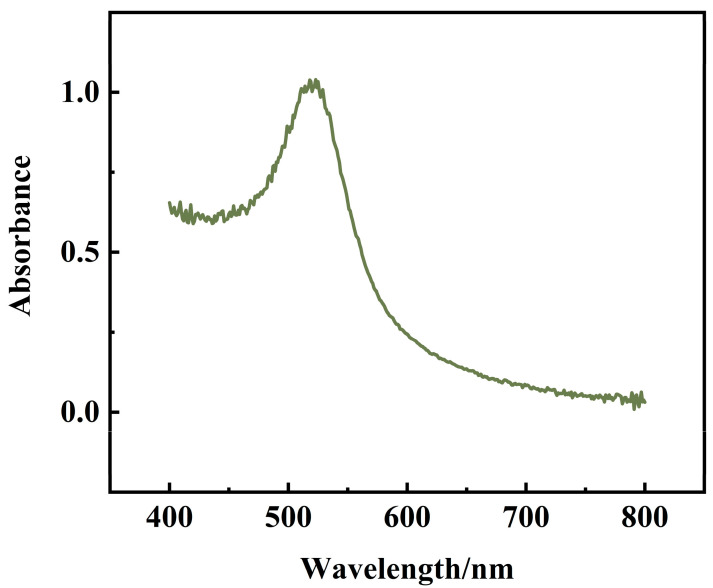
UV-Vis spectroscopy of AuNPs.

The prepared AuNPs were scanned using UV-Vis spectrophotometer in the range of 400–800 nm. As shown in Figure 6, there was an absorption peak at 521 nm. According to the equation of absorption wavelength versus particle size (λmax = 0.3647 D + 515.04, (λmax is the maximum absorption wavelength, D is the particle size)), it can be concluded that the particle size of the prepared AuNPs is 16.3 nm.

The conclusions from the transmission electron micrographs and UV-Vis spectroscopy maps are consistent: the AuNPs prepared in this experiment are uniformly distributed spherical particles with a particle size of 16 ± 1 nm.

### 3.5. Characterization of Capture-Probe-Modified AuNPs

**Figure 7:**
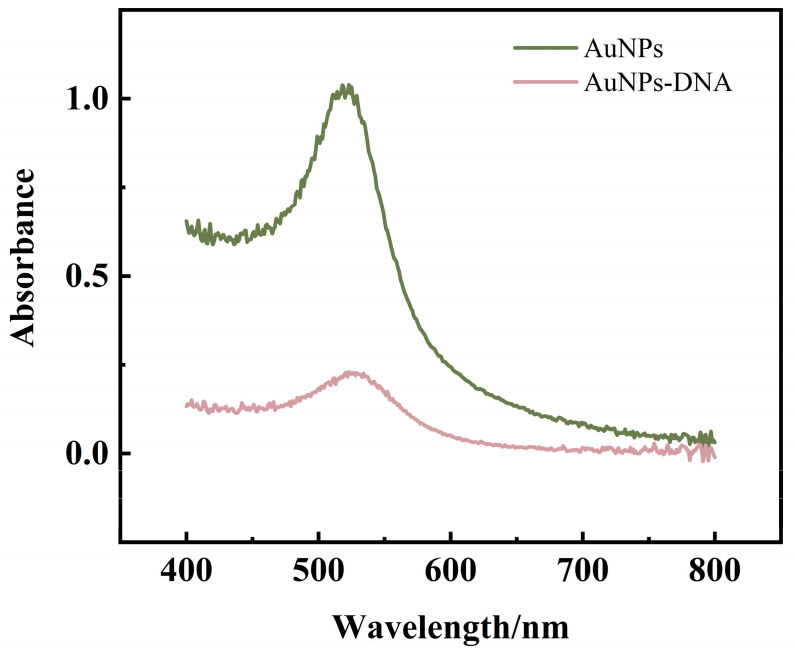
UV-Vis spectroscopy of AuNPs modified by CP.

After CP was ligated with AuNPs, the prepared CP-AuNPs solution was scanned using UV-Vis spectrophotometer in a range of 400–800 nm. From Figure 7, the absorption peak at 521 nm was still present, indicating that the linkage of CP with AuNPs did not cause any change in the particle size of AuNPs, i.e., the modification of the probe did not cause the aggregation of AuNPs. The difference in absorbance between the two substances is mainly due to the fact that the concentration of the CP-AuNPs solution in which the test was performed is lower than that of the AuNPs solution tested.

**Figure 8:**
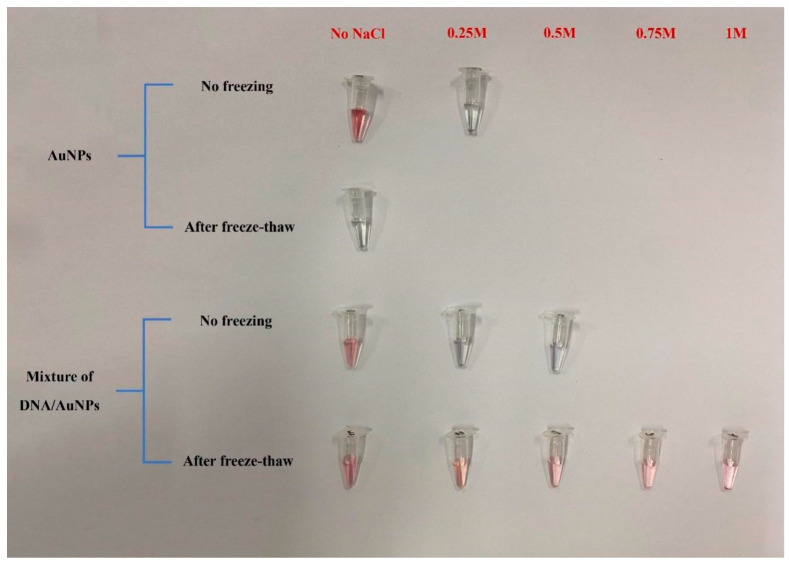
Aggregation of different AuNPs before and after freeze–thaw method.

In this experiment, the AuNPs solution was prepared using the trisodium citrate reduction method, as shown in Figure 8. The prepared AuNPs kept the colloid stable, showing a clear burgundy color owing to the adsorption of negatively charged citric acid ions on the surface before freeze–thawing. However, the adsorption is not strong, and even the addition of a low concentration of salt (0.25 mol/L NaCl) makes the system irreversibly aggregated, and transparent light blue progressively replaces red in the solution. When the AuNPs solution was frozen, the solution color was clear blue and remained light blue after thawing, indicating that irreversible aggregation of AuNPs occurred. This phenomenon is caused by the fact that when frozen, as the temperature decreases, the water in the solution forms ice crystals, which makes the local concentration of gold nanoparticles increase and form their own agglomerates.

After the CP and AuNPs were mixed thoroughly, the aggregation started to occur with the addition of 0.25 mol/L NaCl solution, and the color changed. When adding 0.5 mol/L NaCl solution, the complete aggregation changed to a blue color. The fully mixed CP and AuNPs were frozen at −20 °C for 2 h and thawed at room temperature, and the solution was red, indicating that the solution did not aggregate. Even when 1 mol/L NaCl solution was added, the sample still appeared red, indicating that the mixed solution remained stable without aggregation. This indicates that CP successfully combined with AuNPs and the CP-AuNPs formed a more stable complex after freeze–thawing.

### 3.6. Improving Experimental Conditions

The performance of this biosensor was optimized by using a single variable technique, which also ensured the best detection outcomes. To facilitate error analysis, three replicate experiments were conducted for each experiment.

**Figure 9:**
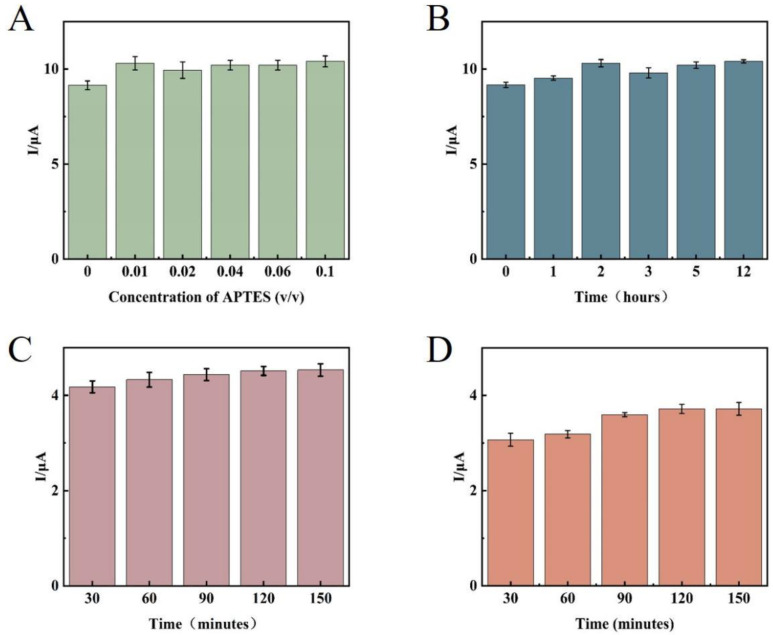
Effects of (**A**) concentration of the APTES; (**B**) APTES reaction time; (**C**) incubation time of AP1 and AP2; (**D**) incubation time of TD.

First, the concentration and time of the APTES-modified glassy carbon electrode were optimized. The analysis results showed that (Figure 9A), under the strict control of water in the external environment, because of the small substrate surface area of the glassy carbon electrode, the APTES molecular weight required to form the self-assembled membrane was small, and 1% concentration of APTES solution could meet the experimental requirements. The the signal value was not significantly enhanced as the concentration increased, so 1% APTES ethanol solution was determined as the best modification concentration for this experiment. After the concentration was determined, the reaction time of APTES was optimized. Theoretically, after the membrane was formed through self-assembly, extending the reaction time did not change the structure and properties of the membrane. However, the reaction time should not be too long because APTES molecules tend to hydrolyze to produce disordered polymers deposited on the substrate surface. From the experimental results (Figure 9B), the signal value remained basically stable after 2 h of system reaction, thus determining 2 h as the modification time of APTES. The signal value stopped changing after 2 h of reaction in this experiment, which employed a 1 μmol/L AP1 and AP2 mixed solution for time optimization. This indicates that AP1 and AP2 completely interacted, and 2 h was found to be the best reaction time (Figure 9C). Since the TD concentrations detected in the experiments were different, a larger concentration of 10 μmol/L (none of the TD concentrations in the subsequent experiments would be larger than 10 μmol/L) was selected for time optimization, and it can be seen from Figure 9D that the 10 μmol/L TD solution reacted completely at 2 h. Therefore, 2 h was determined as the incubation time of the target substance.

### 3.7. Sensor Performance

**Figure 10:**
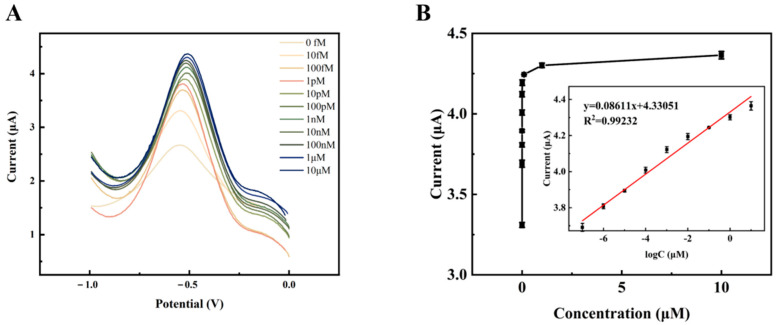
(**A**) Several TD concentrations (1.0 × 10^−14^, 1.0 × 10^−13^, 1.0 × 10^−12^, 1.0 × 10^−11^, 1.0 × 10^−10^, 1.0 × 10^−9^, 1.0 × 10^−8^, 1.0 × 10^−7^, 1.0 × 10^−6^, 1.0 × 10^−5^ mol/L) were detected in the DPV signals. (**B**) Association between DPV intensities and TD concentrations. Insert: calibration curve between the logarithm of TD concentrations and ΔI.

With the optimal experimental conditions obtained above, a gradient of DPV concentration was performed for the target detector (HPV 16). Given as the ideal reaction time, the current response value of the DPV steadily rose with the rise in TD concentration from 1.0 × 10^−14^ mol/L to 1.0 × 10^−5^ mol/L, as shown in Figure 10A. As the amplification grows and expands, more signal molecules [Ru(phen)_3_]^2+^ are inserted into the double helix structure of the DNA, acting as an amplifier of the signal. This suggests that more target DNA is immobilized on the electrode, allowing more long-stranded DNA to be attached to the electrode. A satisfactory linear association between the DPV signal value and the target DNA concentration from 1.0 × 10^−13^ mol/L to 1.0 × 10^−5^ mol/L was found by fitting the linear regression data (Figure 10B), with the regression equation ΔI = 0.08611 log C_TD_+ 4.33051 (R^2^ = 0.9923). The detection limit of this electrochemical biosensor may be estimated using the regression equation LOD = 3σ/S = 1.731 × 10^−16^ mol/L (where the standard deviation of the blank sample set is denoted as σ and the slope of the fitted line in the inset of Figure 10B is denoted as S). Comparing the current study to other previously reported biosensors for the detection of HPV 16 as well as to the prior procedure, it revealed a lower detection limit and a wider linear range (as shown in Table 2). The aforementioned findings imply that this DNA electrochemical biosensor can detect target DNA with excellent sensitivity and a broad linear range.

### 3.8. Sensor Selectivity and Stability

**Figure 11:**
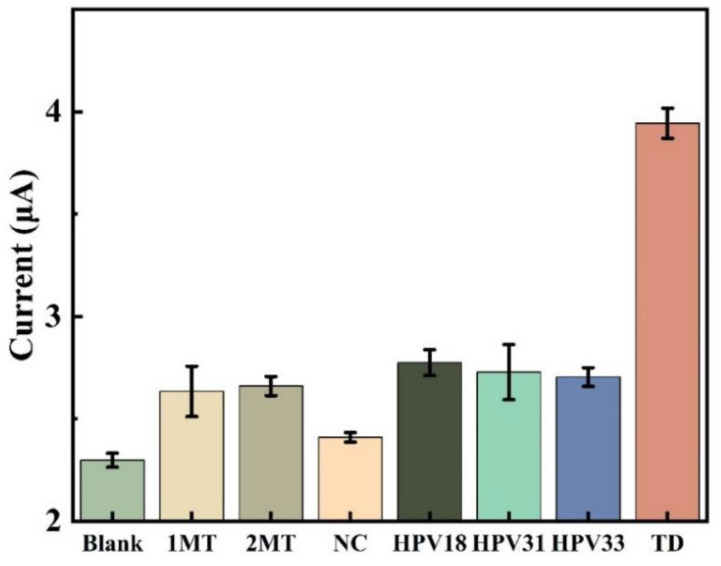
Comparison of DPV reactions for various DNA (NC,2MT, 1MT, HPV33, HPV31, HPV18, TD) and blank sample sets. TD concentration is 1 nmol/L, other sample concentrations are 10 nmol/L.

In this study, the selective expression of this DNA electrochemical biosensor was investigated by comparing sample solutions of eight different DNA sequences, each consisting of three parallel sets of experiments for error analysis. Figure 11 displays how well this sensor selects various targets. Compared to the concentration of other DNA sequences, the concentration of TD chosen for the experiment is ten-times lower. The peak current intensity of the DPV assay in the blank control was only slightly lower than that of the assays containing 2MT, 1MT, NC, HPV 33, HPV 31 and HPV 18, but much lower than that of the TD assay. The low signal value indicates that the sensing system is highly selective for the target HPV 16 and prevents the influence of non-target DNA sequences on the detection results. This indicates that the probe created by the sensor cannot hybridize and pair with other DNA base sequences, and the amplification product cannot connect with the electrode in the absence of the target DNA.

**Figure 12:**
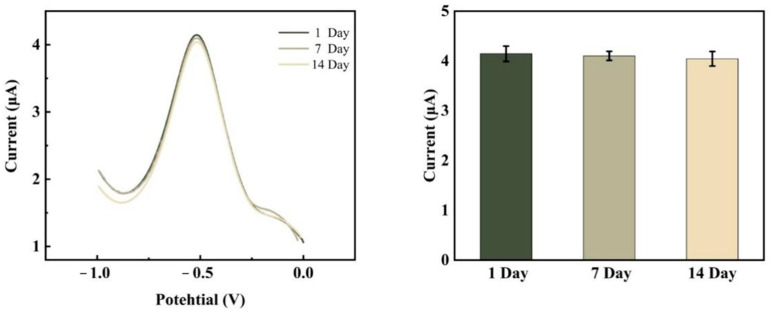
DPV reaction prior to and following the sensor’s placement at 4 °C for 7 days and 14 days, respectively.

The stability of the electrochemical DNA biosensor was also examined under ideal circumstances. The electrochemical tests were conducted on day 1 when the sensor preparation was completed and on days 7 and 14 when it was left at 4 °C. From Figure 12, the current response value decreased by only 1.008% after 7 days and by only 2.420% after 14 days, indicating the good stability of the electrochemical biosensor. The choice of material for the sensor substrate, as well as a more optimal modification process of the electrode surface, led to a further improvement in stability compared to the team’s previous scheme [25].

### 3.9. Detection in Real Samples

The ability of a sensor to detect in challenging environments such as serum refers to the ability of the sensor to accurately detect target substances in complex samples. Serum, as a complex biological sample, contains a variety of biomolecules and chemicals, such as proteins, sugars and lipids, the presence of which may interfere with the sensor’s detection. Therefore, evaluating the detection ability of the sensor in complex environments such as serum is important for its practical application. Recovery tests were performed by adding different concentrations of TD (1, 10 and 100 nmol/L) to the serum solution. The results are presented in Table 3. The recoveries ranged from 98.70% to 100.53%, with relative standard deviations (RSD) of 1.74% to 3.14%. The recovery rate is an important indicator for evaluating the accuracy of the sensor, which indicates the agreement between the sensor’s measurement results and the actual concentration, demonstrating that the sensor is capable of achieving a high degree of accuracy and consistency in complex samples. The relative standard deviation results indicate that the sensor is able to maintain stable detection performance in the presence of interfering substances in the sample and has good anti-interference capability.

## 4. Conclusions

In this experiment, the substrate used is a glassy carbon electrode, and APTES was used to construct the self-assembled membrane on the glassy carbon substrate by covalent bonding to obtain a more stable biosensor system for HPV 16 detection. After placing the sensor for 7 days, the current corresponding value only decreased by 1.008%, and after 14 days, the current response value only decreased by 2.420%, which is more stable. The sensor’s sensitivity is increased by dual-signal amplification by gold nanoparticles and chain amplification reaction. The sensor sensing range is from 1.0 × 10^−13^ mol/L to 1.0 × 10^−5^ mol/L, and the detection limit of the sensor is 1.731 × 10^−16^ mol/L. The ligation of DNA and gold nanoparticles using the freeze–thaw method is simpler and faster compared to the conventional salt aging method. Moreover, the sensor has great detection capabilities in both selective and complex contexts, which has practical application in clinical testing and generates fresh insights for illness detection and diagnosis.

Electrochemical biosensors, as an important analytical and detection technology, still have some needs for continuous innovation and improvement. The application of new materials will provide more options for the design and preparation of electrochemical sensors. The special properties and high specific surface area of nanomaterials have become ideal candidates for electrochemical sensors, and in the future, the further development of nanotechnology will provide more nanomaterials and nanostructures for the construction of high-performance electrochemical sensors. In the future, the development of data processing and intelligent algorithms will provide more possibilities for the interpretation and application of the results of electrochemical sensors, and the large amount of data generated from the experimental results will be reasonably processed and analyzed, and, combined with microfluidic technology and automated instruments, etc., will be intelligently used to achieve more efficient and accurate analysis and detection.

## Figures and Tables

**Table 1:** Sequences of the oligonucleotides used in this study.

Name	Sequence (5′–3′)
Capture probe (CP)	TTTCAATTTTTGGATTAC-SH
Target DNA (TD)	GTAATCCAAAAATTGAAAACTAAGGGTCTGAGGG
Auxiliary probe 1 (AP1)	TACTCCCCCAGGTGCCCCTCAGACCCTTAGT
Auxiliary probe 2 (AP2)	GCACCTGGGGGAGTAACTAAGGGTCTGAGGG
Noncomplementary sequence (NC)	CCTTTTAGTCAGTGTGGAAATCTCTAGCAGTGGC
Single-base mismatch target (1MT)	GTAATCCAA**T**AATTGAAAACTAAGGGTCTGAGGG
Two-base mismatch target (2MT)	GTAATCCAA**TT**ATTGAAAACTAAGGGTCTGAGGG
HPV 18	GTATATTGCAAGACAGTATTGGAACTTACAGAGG
HPV 31	CCAAAAGCCCAAGGAAGATCCATTTAAA
HPV 33	CACATCCACCCGCACATCGTCTGCAAAA

**Table 2:** The proposed sensor is compared with other sensors for the detection of HPV 16.

Dynamic Line Arrange (mol/L)	LOD (mol/L)	Method	Reference
3.50 × 10^−12^–3.53 × 10^−11^	1.750 × 10^−12^	DPV	[29]
5.00 × 10^−10^–1.00 × 10^−7^	1.500 × 10^−10^	DPV	[30]
1.00 × 10^−14^–1.00 × 10^−6^	1.000 × 10^−15^	EIS	[31]
1.00 × 10^−10^–2.00 × 10^−7^	3.000 × 10^−11^	ECL	[32]
1.00 × 10^−13^–1.00 × 10^−6^	5.475 × 10^−16^	DPV	[25]
1.00 × 10^−13^–1.00 × 10^−5^	1.731 × 10^−16^	DPV	This work

**Table 3:** The serum sample was tested for HPV 16.

TD Added (nmol/L)	Total Found (nmol/L)	Recovery (%)	RSD (%)
1.0	0.987	98.70	1.74
10.0	9.985	99.85	3.14
100.0	100.533	100.53	1.95

## Data Availability

Not applicable.
